# Incidental Resolution of Dopamine Agonist Induced Impulse Control Disorder with GLP‐1 Receptor Agonist

**DOI:** 10.1002/mdc3.70704

**Published:** 2026-06-08

**Authors:** Hannah Kelly, Thomas Davis

**Affiliations:** ^1^ Vanderbilt University Medical Center Nashville Tennessee USA

**Keywords:** GLP‐1 receptor agonist, impulse control disorder, dopamine agonist, Parkinson's disease

## Introduction

Parkinson's disease (PD) is a neurodegenerative movement disorder characterized by asymmetric rest tremor, rigidity, bradykinesia, and later postural instability. Patients typically respond well to dopaminergic therapy such as with dopamine agonists (DA). However, a DA side effect to consider is impulse control disorder (ICD). ICD is characterized as the inability to control impulses that can hurt oneself or others. ICD most commonly can present as pathologic gambling, hypersexuality, compulsive shopping, and binge‐eating.^1^ Other than DA dose lowering or discontinuation, there is limited evidence for effective treatments for ICD.

Glucagon‐Like Peptide‐1 receptor agonists (GLP‐1RA) are a class of medications that treat type 2 diabetes and obesity. Research for their use in other disorders is ongoing.^2^, ^3^ To our knowledge, here, we present the first case of resolution of DA‐induced ICD when incidentally treated with a GLP‐1RA.

## Case

A 46‐year‐old man presented for 2 years of slowly progressive stiffness and slowness of his movements without tremor. He did not have major issues with lightheadedness, constipation, visual hallucinations, mood, or memory. His examination showed mild loss of facial expression and decreased speech volume, left greater than right mild rigidity, and bradykinesia. Gait was steady and independent but with decreased left arm swing without retropulsion. Brain MRI was unremarkable.

He was suspected to have Parkinson's disease and trialed on dopaminergic therapy, which provided symptomatic benefit. Due to his young age and lack of non‐motor symptoms, he was started on DAs. He was found to have good motor response on extended release pramipexole 1.5 mg three times daily. On this medication, he began to have frequent thoughts of spousal infidelity, which was atypical for him. Due to concern for ICD, the dose was lowered to 1 mg three times daily and carbidopa/ levodopa extended release capsules 48.75–195 mg three times daily was added for better motor symptom control. However, he did not feel as much motor benefit on this regimen particularly on longer workdays so he increased pramipexole back to 1.5 mg three times daily. Though a plan for DA discontinuation was discussed, he felt his hypersexual thoughts were well‐controlled and that the ICD had transitioned to a compulsory feeling to play pickleball, which he felt was beneficial to his health so opted to remain on this regimen.

But when he returned, he noted his ICD had worsened—his hypersexual thoughts had increased resulting in extramarital affairs hurting his marriage. Additionally, he had new compulsive shopping resulting in $30,000 of excessive clothing spending.

The patient was started on a GLP‐1RA, semaglutide 1 mg weekly, for the treatment of unrelated proteinuria. With these injections, the patient noticed complete cessation of his hypersexual thoughts, which he described as “water being put on a fire.” He did note some mild recurrence of thoughts the day before his next injection was due however the thoughts would again completely resolve following each weekly injection. When he accidentally ran out of his GLP‐1RA, he also felt recurrence of his ICD. He continued on his levodopa formulation and DA with good motor control but he no longer had problems with hypersexuality or compulsive spending while on semaglutide for the past 6 months.

## Discussion

Parkinson's disease typically responds well to dopaminergic therapy like with DAs however their use is somewhat limited by side effects like ICD. ICD has been seen to occur in 1/6 patients on DAs.^4^ Currently, there is limited treatment for DA‐induced ICD with the primary approach being DA discontinuation or dose taper.^5^ Though pharmacotherapy with antipsychotics, anti‐seizure medications, and naltrexone have been considered for ICD, consensus guidelines recommend against their use given lack of evidence.^6^ Psychological interventions like cognitive behavioral therapy and use of selective serotonin reuptake inhibitors can occasionally be helpful for ICD but clinical guidelines about the most effective approach remain unclear and further clinical trials are needed.^7^

Ongoing and extensive research is looking at the utility of GLP‐1RAs in not only metabolic disorders but also neuropsychiatric diseases.^8^ Semaglutide has been shown to block the typical release of dopamine from alcohol in the brain's reward system in rodents,^9^ which suggests a mechanism for GLP‐1 agents to modulate addictive behaviors. We theorize this GLP‐1RA mechanism of action in ICD as well. A recent meta‐analysis of GLP‐1RAs in JAMA Psychiatry noted reduced nicotine and alcohol cravings in people on GLP‐1RAs.^10^ This review suggests that GLP‐1RAs may have a direct brain effect with signaling in the mesolimbic reward area contributing to mental health benefits,^10^ supporting our theorized mechanism of action of GLP‐1RAs in ICD.

Our case of DA‐induced ICD incidentally treated with a GLP‐1RA suggests a possible novel way to treat ICD, which currently has limited treatment options. Though more research is needed, this association should be kept in mind for patients suffering from DA‐induced ICD who have other indications for GLP‐1RAs.

This research did not require IRB approval as no experiments were performed on human or animal subjects. CARE checklist completed and included. Written informed consent‐to‐disclose form has been reviewed and signed by the case patient. We attest that authorization signed by the patient has been obtained in compliance with any laws regarding patient authorizations relating to the use or disclosure of protected health information of the jurisdiction(s) to which the patient and the physician are subject including, if applicable, the United States Health Insurance Portability and Accountability Act of 1996 (“HIPAA”). We confirm that we have read the Journal's position on issues involved in ethical publication and affirm that this work is consistent with those guidelines.

## Disclosure

All authors have read the manuscript, the paper has not been previously published, and it is not under simultaneous consideration by another journal. There was no ghost writing by anyone not named on the author list. The authors have no financial or competing interests to disclose.

## Data Availability

Data sharing not applicable to this article as no datasets were generated or analysed during the current study.
